# Nonstandard working schedules and health: the systematic search for a comprehensive model

**DOI:** 10.1186/s12889-015-2407-9

**Published:** 2015-10-23

**Authors:** Suzanne L. Merkus, Kari Anne Holte, Maaike A. Huysmans, Willem van Mechelen, Allard J. van der Beek

**Affiliations:** Research group Work and Safety, International Research Institute of Stavanger, PO Box 8046, 4068 Stavanger, Norway; Department of Public and Occupational Health, EMGO Institute for Health and Care Research, VU University Medical Center, PO Box 7057, 1007 MB Amsterdam, The Netherlands; Body@Work TNO VUmc, Research Center on Physical Activity, Work & Health, VU University Medical Center, Amsterdam, The Netherlands

**Keywords:** Recovery after work, Shift work, Night work, Extended working hours, Extended working weeks, Theory

## Abstract

**Background:**

Theoretical models on shift work fall short of describing relevant health-related pathways associated with the broader concept of nonstandard working schedules. Shift work models neither combine relevant working time characteristics applicable to nonstandard schedules nor include the role of rest periods and recovery in the development of health complaints. Therefore, this paper aimed to develop a comprehensive model on nonstandard working schedules to address these shortcomings.

**Methods:**

A literature review was conducted using a systematic search and selection process. Two searches were performed: one associating the working time characteristics time-of-day and working time duration with health and one associating recovery after work with health. Data extracted from the models were used to develop a comprehensive model on nonstandard working schedules and health.

**Results:**

For models on the working time characteristics, the search strategy yielded 3044 references, of which 26 met the inclusion criteria that contained 22 distinctive models. For models on recovery after work, the search strategy yielded 896 references, of which seven met the inclusion criteria containing seven distinctive models. Of the models on the working time characteristics, three combined time-of-day with working time duration, 18 were on time-of-day (i.e. shift work), and one was on working time duration. The model developed in the paper has a comprehensive approach to working hours and other work-related risk factors and proposes that they should be balanced by positive non-work factors to maintain health. Physiological processes leading to health complaints are circadian disruption, sleep deprivation, and activation that should be counterbalanced by (re-)entrainment, restorative sleep, and recovery, respectively, to maintain health.

**Conclusions:**

A comprehensive model on nonstandard working schedules and health was developed. The model proposes that work and non-work as well as their associated physiological processes need to be balanced to maintain good health. The model gives researchers a useful overview over the various risk factors and pathways associated with health that should be considered when studying any form of nonstandard working schedule.

## Background

From a societal perspective, the continuous availability of staff outside the 9–17 h working day is necessary in some sectors. This requirement is met by nonstandard working schedules, i.e. any schedule other than the regular 40 h working week (8 h day work, Monday to Friday). These schedules include shift work (6 pm–6 am), extended working hours (>8 h), extended working weeks (>5 days), on-call duties, weekend work, and combinations thereof, for example found in health care, and the trucking, airline, mining, and offshore industries [1–6]. It is estimated that 51 % of the European work force works some form of nonstandard working schedule [7].

From a worker’s perspective, nonstandard working schedules have been associated with negative health effects. Working in extended weeks leads to increased fatigue [5, 8], while working extended hours has an increased risk for sleep disturbances, need for recovery, and coronary heart disease [9–12]. Although the evidence is not always conclusive, shift work is suggested to increase the risk for fatigue, sleepiness, shift work sleep disorder, gastro-intestinal problems, diabetes type II, cancer, and cardiovascular disease [9, 13–18].

Models and theories describing the association between nonstandard working schedules and the health effects described above are deeply rooted in shift work theory. Models on shift work traditionally have a biomedical perspective, focusing on disrupted sleep/wake cycles and circadian rhythms [19–21] and, thus, on the effects of the time-of-day that work is scheduled. However, many nonstandard schedules found in society and studied in research combine shift work with extended working hours, e.g. durations of 10–24 h, and current shift work models do not provide insights into whether and how shift duration, next to time-of-day, may play a role in the onset of health complaints. Theories on extended working time duration may shed a light on this matter, with their psychophysiological perspective, focussing on increased activation due to long exposure to work demands [22, 23]. Integrating shift work models, i.e. models on time-of-day, with models on working time duration would better reflect the diversity of schedules found in society and studied in research. For these schedules, the integration would lead to an increased understanding of how time-of-day and working time duration—separately and jointly—can contribute to the onset of health complaints.

Additionally, models on shift work do not include the role that rest periods play in health, although the European Commission’s Working Time Directive emphasises the importance of ‘adequate rest periods’ for health maintenance [24]. Recovery, that takes place during rest periods, is considered to be important for health maintenance and has been defined as the return to and stabilisation of psychophysiological systems at a baseline level of activation in the absence of work demands [25]. Shift work and extended working hours have been associated with a higher need for recovery [9]. It is hypothesised that nonstandard working schedules that include shift work, extended working hours, and/or extended working weeks may impede the recovery process [26] and may over time predispose the individual to negative health effects [25, 27]. Recovery has been put forward as a protective mechanism in models on shift work and extended working hours [21, 22, 28]. Integrating models on recovery with shift work models, i.e. models on time-of-day, and models on working time duration could increase the understanding of what makes a rest period ‘adequate’ for those working in nonstandard schedules and how their health could be protected by rest periods.

The overall purpose of this paper is to develop a comprehensive model on nonstandard working schedules and health that better reflects the diversity of schedules found in society and that includes the health effects of rest periods. This was done by combining models on shift work, i.e. the time-of-day work is scheduled, with models on working time duration and recovery.

## Methods

A literature review was conducted to gain an overview of models that associated 1) the working time characteristics time-of-day (such as in shift work) and working time duration (such as in extended working hours) with health; and 2) recovery after work with health. Data extracted from the models formed the basis for the development of a comprehensive model describing the association between nonstandard working schedules and health.

The literature review was conducted using a systematic search and selection process. Nonstandard working schedules were defined broadly as any schedule other than the traditional Monday to Friday working week consisting of five consecutive 8 h working days. Excluded from this review were flex-work, part-time work, and self-rostering practices. This was done to provide an overview of the health effects of full-time work, as well as an overview of health maintenance suggestions for schedules that do not permit employee working time control. In accordance with Costa [29], health was defined according to the World Health Organisation’s definition as “a state of complete physical, emotional, and social well-being and not merely the absence of disease or infirmity” [30]. Models, theories, frameworks, mechanisms, pathways, and constructs were included in the search process and are summarised as ‘models’ in the remainder of the paper.

### Search methods

#### Sources

Systematic searches were conducted in three databases from inception to 31^st^ January 2014 to retrieve peer-reviewed articles, books, and PhD dissertations. Searches were conducted in Medline with EBSCOhost, in PsycINFO with Ovid, and in EMBASE with EMBASE.com. Additionally, reference lists of relevant articles were hand-searched, and references were included by the snowball method.

#### Search strategy

A search specialist at the VU University Medical Center Library in Amsterdam, the Netherlands, was consulted for developing the search strategy. Two search strategies were developed: one for models associating the working time characteristics time-of-day and/or working time duration with health, and one for models associating recovery after work with health. The controlled vocabulary thesauri from the databases were used to retrieve useful search terms: Medical Subject Headings of MEDLINE, Major Subject Headings of PsycINFO, and the EMTREE of EMBASE. The Boolean operators AND, OR, and NOT, as well as the proximity operators NEXT, ADJ, and NEAR, were incorporated into the search terms. The search strategy developed for Medline is given in Table 1. Similar strategies were used for PsycINFO and EMBASE. Search results were restricted to the English language.

**Table 1:** Search strategy used for Medline via EBSO*host*

Limits: Human, English language, Booelan search, Abstract
Time-of-day (i.e. shift work) and working time duration and health
(MH “Work Schedule Tolerance”) OR (MH “Chronobiology Disorders”) OR (MH “Sleep Disorders, Circadian Rhythm+”) OR AB ((Shiftwork) OR (Nightwork) OR (Nightshift) OR (shift* N2 work*) OR (night* N2 work*) OR (night* N2 shift*) OR (Night* N2 schedul*) OR (Shift* N2 system*) OR (Shift* N2 schedul*) OR (Shift* N2 rotat*) OR (Shift* N2 pattern*) OR (Evening N2 work) OR (Evening N2 shift*) OR (Morning N2 work) OR (Morning N2 shift*) OR (irregular* N2 shift*) OR (irregular* N2 work* N2 hour*) OR (irregular* N2 schedule) OR (nonstandard N2 schedul*) OR (Work* N2 time N2 arrangement*) OR (Long N2 work* N2 hour*) OR (hour* N2 long) OR (hour* N2 extend*) OR (Extend* N2 week*) OR (Compress* N2 hour*) OR (Compress* N2 week*) OR (weekend N2 work*) OR (Sleep N2 wake N2 cycle) OR (circadian N2 stress) OR ((circadian N2 rhythm*) AND work))
AND (MH “Health+”) OR (MH “Pathological Conditions, Signs and Symptoms+”) OR AB ((Health) OR (Occupational N2 health) OR (Illness*) OR (Impairment*) OR (Health N2 outcome*) OR (Disease) OR (Health N2 complaint*) OR (well N2 being))
AND (MH “Models, Theoretical”) OR (MH “Models, Biological+”) OR (MH “Models, Psychological”) OR AB ((Model*) OR (theor*) OR (construct) OR (framework) OR (mechanism*) OR (pathway*))
Recovery after work and health
(MH “Relaxation+”) OR (MH “Fatigue+”) OR AB ((Recuperate) OR (Recuperation) OR (Rest) OR (Resting) OR (Recovery) OR (Recovery N2 after N2 work) OR (Recovery N2 from N2 work) OR (unwind*) OR (nonwork) OR (non-work))
AND (MH “Work+”) OR (MH “Workload”) OR AB ((work* N2 schedul*) OR (Work* N2 time N2 arrangement*) OR (Work* N2 arrangement*) OR (Long N2 work* N2 hour*) OR (hour* N2 long) OR (hour* N2 extend*) OR (Week* N2 work* N2 hour*))
AND (MH “Models, Theoretical”) OR (MH “Models, Biological+”) OR (MH “Models, Psychological”) OR AB ((Model*) OR (theor*) OR (construct) OR (framework) OR (mechanism*) OR (pathway*))

#### Selection process

Determination of eligibility for inclusion was done by one reviewer (SLM) at two levels. At the first level, references were screened for eligibility based on title and abstract. At the second level, full text articles were retrieved and evaluated for those titles and abstracts that seemed eligible for inclusion or for which eligibility was unclear, e.g. due to too little information.

### Inclusion and exclusion criteria

Articles were included if they met all of the following criteria:

1. Description of a framework, theory, model, construct, mechanism, pathway, or model:
  on time-of-day and/or working time duration and health (first search)
  on recovery after work and health (second search)
2. A. For time-of-day and/or working time duration, any (combination) of the following:
  shift work (hours worked 6 pm–6 am, including early morning, evening, and night work)
  extended working hours (>8 h/day)
  extended working weeks (>5 days/week)
  on-call duties
  weekend work
  B. For recovery after work, one of the following characteristics:
  psychological unwinding (recovery experiences, need for recovery, improvement in fatigue)
  physiological unwinding (e.g. recovery in neuroendocrine parameters, cardiovascular parameters, body temperature)
  sleep as recovery activity
3. Population: humans and a working population
4. Source: primary research, secondary research, books, and theses/dissertations

Articles were excluded if one of the following criteria were met:

1. A model centred round:
  part-time work, flex-work, self-rostering
  the onset of fatigue (as apposed to recovery from work-related fatigue)
  statistical/mathematical methods only
2. Outcomes of models were not health but:
  quality of life, work performance, or job satisfaction
3. Source:
  conference proceedings

### Data extraction and data preparation

From each model, data was gathered on working time and on other work schedule characteristics; on all pathways associating these characteristics to health outcomes; and on the health outcomes. The data was prepared for development of a comprehensive model by 1) identifying and grouping working time characteristics, and 2) identifying and grouping similar health pathways.

## Results

### Search and selection

**Fig. 1:**
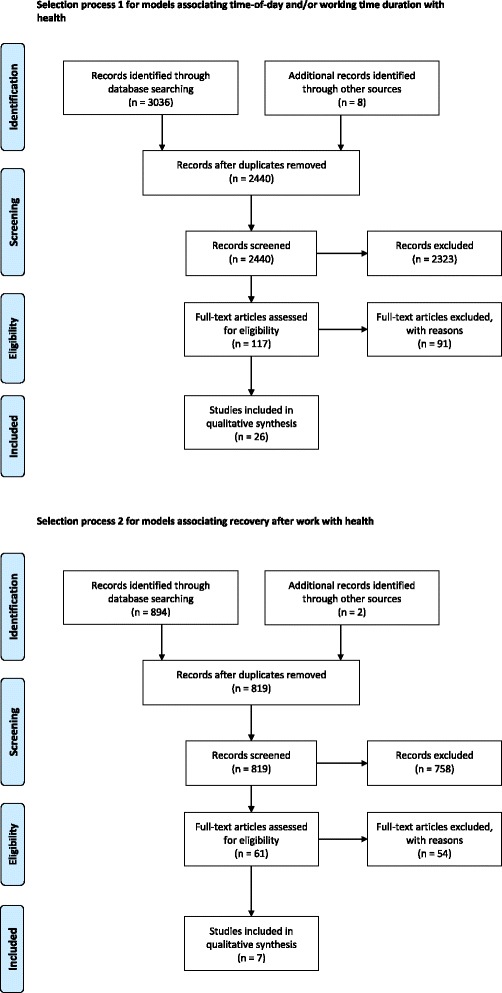
An overview of the number of articles found, screened, and included in the review

Figure 1 gives an overview of the results from the selection processes for the two searches. The first search strategy for models associating time-of-day and/or working time duration with health resulted in 3044 references. After duplicates were removed, 2440 titles and abstracts were screened. In total, 117 full-text papers were read; finally, 26 articles were included into the review. The most frequent reasons for exclusion were the absence of a model in combination with the focus on other topics related to health (e.g. management strategies) (*n* = 918), and the focus on non-working populations (e.g. patient populations) (*n* = 416).

The second search strategy for models on recovery after work and health resulted in 896 references. After the removal of duplicates, 819 titles and abstracts were screened. In total, 61 full text articles were read; finally, seven articles were included in the review. Most frequently, articles were excluded because they did not present a model and did not focus on a working population (e.g. recovery from exercise or alcoholism) (*n* = 684).

### Summary of the included articles

From the 26 articles found on models on time-of-day and/or working time duration (Table 2), 29 models were identified: 23 articles described one model and three articles included two models [31–33]. However, six articles summarised or only slightly extended previous models [33–38], which led to 22 distinctive models that described the association between a form of nonstandard working schedule and health.

**Table 2:** Overview of identified models associating health with time-of-day (such as in shift work), working time duration (such as in extended working hours), and nonstandard working schedules (where time-of-day and working time duration are combined)

Study	Schedule	Model summary	Health outcome
1. Rutenfranz [52]	Shift work	The association between objective stress (phase shifting of work and sleep) and strain (lowered well-being and disease) is mediated by intervening variables(e.g. personality, physiological adaptability). The intervening variables determine whether a person can cope with shift work or whether lowered well-being may develop into disease.	Complaints and disease
2. Haider [31]	Shift work	1. Model by Åkerstedt et al. (1977). Adjustment of the circadian rhythm to working hour requirements may lead to complaints and social role conflicts. Depending on exposure, health state, and personality, this may in turn lead to poor attitudes, absenteeism, and illness.	1. Digestive problems, illness, absenteeism
		2. Destabilisation hypothesis from Kundi et al. (1979). Adaptation to shift work is central in the maintenance of shift worker health, accomplished by a stable interaction between family life, sleep behaviour, and attitudes towards shift work. Personal factors, work situation, and social environment modify the interaction. An unstable interaction may, over time via a sensitisation phase, lead to health complaints (accumulation phase).	2. General health problems
3. Folkard [20]	Shift work	Shift work may affect three interrelated life domains: the biological domain (circadian disruption), medical domain (short-term health consequences), and social domain (social and family activities). Symptoms in these domains may lead to general feelings of malaise in susceptible individuals.	General feelings of malaise
4. Monk [86]	Shift work	Shift work is seen as a source of stress, due to a reversed sleep/wake cycle, that must be coped with. A triad of coping factors—biological clock, social/domestic, and sleep— are described that influence tolerance to shift work.	Sleep and stomach complaints, malaise
5. Kundi [37]	Shift work	Destabilisation theory of shift work, adapted from Kundi et al.(1979) and Haider et al. (1981). The model describes a complex dynamic interaction between three activity spheres (work, family, and recreation) that need to stay in equilibrium to preserve health (adaptation phase). Personality traits, attributions of the social environment, and work situation play a mediating role between the interacting spheres. An unstable interaction between the spheres may, over time, lead to health complaints (accumulation phase) via sensitisation phase.	Health state/complaints
6. Knutsson [43]	Shift work	Three inter-related pathways leading from shift work to coronary heart disease are described:	Coronary heart disease
		1. Mismatch of circadian rhythms.	
		2. Psychosocial factors lead to stress-induced changes, e.g.lipoprotein disturbances.	
		3. Behavioural and life-style changes (coping) (e.g. diet, smoking).	
7. Knutsson & Bøggild [38]	Shift work	Adjusted slightly from Knutsson [43]; it adds three ways in which circadian rhythms can be mismatched:	
		1. A phase shift of circadian rhythm relative to the day-night cycle.	
		2. Desynchronisation of different internal body rhythms.	
		3. Reduced rhythm amplitude.	
8. Olsson [53]	Shift work	Model based on the transactional psychological theory of stress and coping. Stress occurs when there is an imbalance between the person’s resources and the appraised demands from the environment (occupational shift work stressors, non-occupational stressors). Passive coping styles may lead to poor health when stressors exceed resources. Active coping styles maintain health. Personal factors influence the balance between appraisal, stress, and coping.	Poor health: mental load, symptoms, absence, well-being
9. Tepas & Mahan [39]	Night work	Night work induces acute sleep loss that accumulateds to ‘total acute sleep loss’ with consecutive shifts. Continued night work over the years may lead to chronic sleep deprivation, which in turn can lead to biological deficiency and medical disorders. Shift work tolerant workers are those able to make suitable changes in lifestyle and values.	Medical disorders, sickness and death
10. Folkard [51]	Shift work	Shift system features, influenced by individual and situational differences, may disturb biological rhythms, sleep, and family/social life. These disturbances may affect mood. Success of coping strategies determine whether acute effects develop into chronic mental health problems, which in turn may develop into negative physical health.	Chronic mental & physical health
11. Tepas [36]	Shift work	Sequential Austrian life span model from Kundi et al. (1979) and Haider et al. (1981). Development of health effects occurs in 4 sequential phases: adaptation phase (0–5 years), sensitisation phase (5–20 years), accumulation phase (20+ years), disease manifestation phase (40+ years). Situational and biological factors may have a stabilising or destabilising effect on each phase. Shift work drop-out may occur at each stage depending on individual stress tolerance and coping development.	Disease
12. Smith & Barton [54]	Shift work	Appraisals of controllability (shift work locus of control) together with actual control over working hours influence situational control, which in turn plays a moderating role in the stress–strain process. External stress arises from working the shift system and internal strain from attempts to cope with the shift system. Reduced situational control may lead to negative health effects and absenteeism.	Absenteeism
13. Barton [34]	Shift work	Same as Folkard [51].	
14. Richardson & Maly [33]	Shift work	Two theories were briefly described that may increase health risk:	Shift work sleep disorder
		1. Chronic circadian disruption, i.e. chronic disruption of normative physiological processes.	
		2. Chronic sleep deprivation, with proximal causes such as heightened exposure to behavioural risk factors.	
15. Smith [44]	Shift work	Individual and situational variables influence the development of sleep, social, and domestic disturbances. The disturbances lead to coping behaviour (active or passive) to handle the stress associated with the disturbances. When coping is unsuccessful, it may lead to short-term effects (decreased emotional and physical well-being) which in turn may result in chronic health problems.	Short-term: fatigue.
			Chronic: digestive and cardiovascular symptoms
16. Perrucci [49]	Shift work	Schedule and job demands are stressors that predict negative health effects. Timing and duration of work and non-work are important: they may negatively (e.g. circadian disruption) or positively (e.g. more time off) influence health. The predictor variables are moderated or mediated by family and work place variables. Demographic and personality variables (e.g. shift work tolerance) independently influence predictor variables.	Physical symptoms
			Mental health and well-being
17. Reinberg [50]	Shift work	The Dian Circadian Model is described. Shift work and physical and/or psychological workload may lead to circadian disruption in some, but not in all individuals (euchronism). Circadian disruption may be present with clinical symptoms (dyschronism) or without clinical symptoms (allochronism). These inter-individual differences are due to genetic differences. Long-term exposure to circadian disruption may sensitise the body, leading from allochronism to dyschronism at a later stage.	Clinical symptoms
18. Puttonen [21]	Shift work	Shift work may lead to circadian stress by disturbing circadian rhythms. Circadian stress is psychosocial stress (e.g. recovery), behavioural stress (e.g. sleep), and/or physiological stress (e.g. inflammation). These three stresses influence each other and may lead to other disease conditions (e.g. atherosclerosis) that precede cardiovascular disease. Physiological stress may directly lead to cardiovascular disease; so may other disease conditions.	Cardiovascular disease
19. Antunes [42]	Shift work	The cause of obesity is a complex interplay between genetic, environmental, psychobehavioural, endocrine, and metabolic factors. Shift work leads to desynchronisation of work, social, and eating patterns, which may cause desynchronisation between central and peripheral oscillators. This in turn may cause weight gain by:	Obesity
		1. Lower metabolic efficiency when eating at night due to gene expression at the ‘wrong’ time of day.	
		2. Fat production in adipose tissue by increased sympathetic output due to stress, job strain, and psychosocial factors.	
		3. Altered glucose and lipid homeostasis due to light at night.	
		4. Lifestyle changes, e.g. reduced physical activity.	
20. Fritschi [19]	Shift work	Shift work may lead to one or more of these mechanisms:	Cancer
		1. Desynchronisation between central and peripheral oscillators causing physiological disruptions and intra-cellular disruptions.	
		2. Light at night may suppress melatonin excretion and thereby reduce its anti-carcinogenic effects.	
		3. Sleep disruption may cause stress axis activation and immune suppression.	
		4. Lifestyle disturbances may lead to negative lifestyles and metabolic changes.	
		5. Less sunshine for night workers may decrease production of Vitamin D and reduce its anti-carcinogenic effects.	
21. Kivimäki [45]	Shift work	Shift work may cause desynchronisation between central and peripheral oscillators and trigger “a cascade of biological changes that have potential diabetogenic effects”. Shift work may also lead to poor or insufficient sleep. Desynchronisation between central and peripheral oscillators and poor or insufficient sleep affect each other, and may lead to insulin resistance and weight gain. This in turn may lead to diabetes type 2.	Diabetes type 2
22. Dickerman & Lui [35]	Night work	Model of Fritschi et al. [20]. Review on evidence of light at night among female nurses working night shifts.	Breast cancer
23. Vallières & Bastille-Denis [40]	Night work	Psychobiological model. Night work may disrupt sleep regulation that leads to shift work sleep disorder. Sleep regulation is controlled by a complex interaction between circadian rhythm and sleep homeostasis, in which adjustment capacity (plasticity) and involuntary processes (automaticity) play a central role. Subsystems protect plasticity and automaticity; these include physiological (e.g. chronotype) and cognitive de-arousal (e.g. no intrusive thoughts), as well as stimulus control (e.g. sleep habits) and facilitation of daytime sleep (e.g. low job stress).	Shift work sleep disorder
24. Haines [41]	Extended working hours	Extended working hours are not a stressor; rather, they are a parameter for duration of physical or mental effort. Therefore, the association between extended working hours and psychological distress is mediated by increased psychological work demands and increased decision latitude.	Psychological distress
25. Caruso [22]	Nonstandard working schedules	The paper describes the Framework for Study of Undesirable Impacts of Long Work. Extended working hours, together with other schedule characteristics, lead to longer exposure to job demands, and reduce the time for recovery and sleep. This may lead to acute effects and chronic illnesses. Worker and job characteristics moderate the associations between time for recovery and acute effects, and between acute effects and chronic illnesses. Chronic illnesses may increase vulnerability to acute effects.	Acute effects and Chronic illnesses
26. Steinmetz & Schmidt [32]	Nonstandard working schedules	1. Sequence model: The effect of job stressors and working time on health outcomes is mediated by sleep quality and chronic fatigue.	1. Gastro-intestinal, cardio-vascular and musculo-skeletal complaints
		2. General strain factor model: The effect of job stressors and working time on health is mediated by a general strain factor. A general strain factor is a common factor underlying the simultaneous expression of various health constructs and it is explained by the process of sensitization of cognitive, emotional, and somatic systems. This model is a better fit to the study data than the sequence model.	2. Somatic complaints, chronic fatigue, sleep quality

Of these 22 models, three models (two of which were described in one article) combined the working time characteristics time-of-day and working time duration [22, 32]. Eighteen models focussed on time-of-day only, of which two specifically focussed on night work [39, 40]. One model was about working time duration [41].

Various pathways contributing to ill health as well as those protective of ill health were identified in the models (Table 2). The models on working time duration and those combining time-of-day with working time duration described sleep and stress-related pathways. The models on time-of-day included a diversity of pathways that could be categorised according to circadian adjustment (circadian disruption and (re-)entrainment), sleep (sleep deprivation and restorative sleep), and activation and recovery.

The health outcomes described by the three models that combined time-of-day with working time duration included somatic complaints (e.g. gastrointestinal, cardiovascular, musculoskeletal), chronic fatigue, sleep problems, stress, discomfort, dysfunction, and cancer [22, 32]. Models on time-of-day included general outcomes, such as complaints [37], disease [36], and general feelings of malaise [20], as well as more specific outcomes such as shift work sleep disorder [40], obesity [42], cardiovascular disease [21, 43, 44], diabetes [45], and cancer [19]. The model on extended working hours had psychological distress as health outcome [41].

Seven different models were found in the articles on recovery after work (Table 3). Five models were about regular day work, while two models were about nonstandard working schedules [21, 22]. The latter two models were also found in the first search for models on time-of-day and working time duration. Health outcomes included general health impairment [23, 25, 46], cardiovascular disease [21], psychological and energetic state [47], and somatic symptoms [48].

**Table 3:** Overview of identified models on recovery after work and health

Study	Schedule	Model summary	Health outcome
Meijman & Mulder [25]	Regular day work	Effort recovery model is described. Mental and physiological activation due to effort expended at work will lead to normal load reactions. These reactions are adaptations to work demands and are short-term physiological, behavioural, and subjective reactions. They are reversible through recovery. Insufficient recovery may lead to negative health effects that are structural changes and can be irreversible.	Negative health effects: Losses of function, health impairment, or illness.
Sluiter [46]	Regular day work	Cumulative process model of stressors, recovery, and health. When job demands exceed the person’s capacity, increased psychophysiological reactivity may lead to a cascade of increased need for recovery and fatigue, and short-term and long-term health effects. Sufficient recovery may counteract these effects. Four timeframes are given in which this may occur: microrecovery (pauses during work that last a few minutes), mesorecovery (a break of 10 min to 1 h after a task), metarecovery (the time between two working days, shifts, or working periods), and macrorecovery (begins two days after work, e.g. vacation).	Health complaints Diseases
Caruso [22]	Nonstandard working schedules	See Table 2	
Geurts & Sonnentag [23]	Regular day work	Two supplementary models are described:	Health impairment
		1. Effort-recovery model: See Meijman & Mulder [25].	
		2. Allostatic load model: Physiological systems are activated under stress and effort at work. When repeated or prolonged they may lead to disturbances in homeostatis and allostasis. This in turn may lead to wear and tear of the body and brain.	
Demerouti [47]	Regular day work	A model on daily recovery after work. Negative strain during work will continue into the home environment where it is influenced by home demands and home resources. The association between strain and psychological and energetic states at bedtime is moderated by the recovery potential of activities. States at awakening are influenced by sleep.	Psychological & energetic state
Puttonen [21]	Shift work	See Table 2	
Biron [48]	Regular day work	The association between need for recovery and sickness absence is mediated by supervisor and co-worker support, and partially mediated by somatic symptoms.	Sickness absence, somatic symptoms

### A comprehensive model on nonstandard working schedules and health

From the literature review it was evident that the working time characteristics time-of-day and working time duration have already been integrated in three models found in two articles [22, 32]. These models addressed major shortcomings of shift work models by adopting a multidimensional approach to working hours and one model included the effects of recovery on health [22, 32]. However, we believe that these models were not sufficiently able to describe the pathways associating nonstandard working schedules with health, as they either lightly touch upon or do not at all mention the central pathway of circadian disruption relevant to the time-of-day work is scheduled [22, 32]. The comprehensive model developed in the present paper built on the strengths and addressed the weaknesses of these three models [22, 32].

**Fig. 2:**
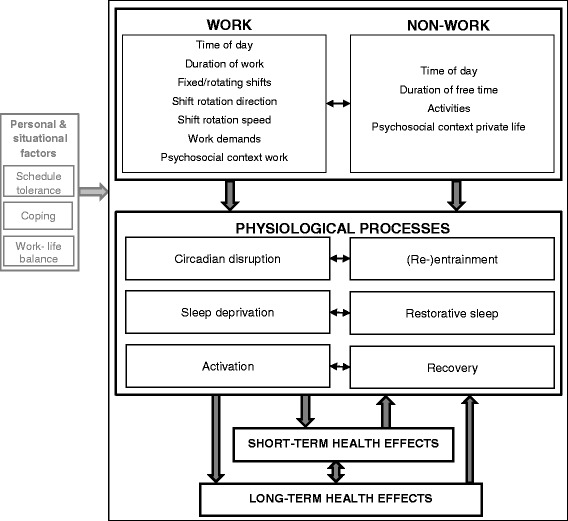
A comprehensive model on nonstandard working schedules and health

The comprehensive model presented in Fig. 2 proposes that work and non-work as well as their associated physiological processes need to be balanced to maintain good health. The concepts underlying work and non-work came from models on time-of-day, working time duration, and recovery [19, 22, 23, 32, 40, 42, 44, 47, 49–51]. From the models on time-of-day and/or working time duration, pathways leading to health complaints were extracted, which led to three pathways: circadian disruption [19–21, 31, 33, 40, 42, 43, 45, 49, 50, 52], sleep deprivation [19–22, 32, 33, 37, 39, 40, 43–45, 52], and increased and/or sustained activation [19, 21, 31, 32, 36, 41, 43, 44, 49, 52–54]. All of which are considered stressors for the body [21, 55]. From the models on recovery as well as the models on time-of-day and/or working time duration, recovery was extracted as a compensatory physiological process for increased and/or sustained activation [21–23, 25, 46–49]. From the models on time-of-day and/or working time duration, the compensatory processes of restorative sleep [39] and circadian (re-)entrainment [40, 42, 52] were retrieved. In the following sections, the new comprehensive model is described and supported by knowledge from the models found in the literature review (references up to [55]) and by some empirical evidence (references beyond [55]).

#### Work and non-work

A large variety of nonstandard working schedules exists, and with that comes an equally large variety of ways to regulate the balance between work and non-work. Work refers to the time-period in which a person invests mental and physical effort into performing tasks in order to acquire an income. Assimilated from the models in the review, important overarching-terms for work relate to working time characteristics and to work characteristics. The working time characteristics are: time-of-day, working time duration, fixed/rotating shifts, and shift rotation direction and speed. The work characteristics are: work demands and psychosocial context of work [22, 32, 40, 42, 44, 48–51]. The overarching terms for the working time characteristics are comparable to the ‘dimensions of working time patterns’ labelled by Härmä et al. [56]. Non-work refers to the time-period in which a person pursues personal, family, and social activities and may recover from and accommodate to working at nonstandard hours. For nonstandard working schedules, important overarching-terms for non-work include time-of-day, duration of free time, leisure time activities, and psychosocial context of the private life [19, 22, 37, 40, 42, 44, 47, 49].

Certain timeframes apply to all nonstandard schedules in which different factors of work and non-work play a role [46, 49]. These timeframes are the “working day or shift” and “work schedule”.

Firstly, of importance for the working day or shift are: the time-of-day that a person is scheduled to work and the duration of the working day or shift [32]. The time-of-day is related to the light/dark cycle. As a diurnal species, humans function best when they are awake during the day and asleep during the night. Night work leads to changes in the sleep/wake pattern, to exposure to light at night [19, 35], and to an altered timing of food intake [42, 45]. Early morning and evening shifts are associated with similar changes, although these changes are less pronounced than those in night shifts. The duration of a working day or shift determines the duration of effort expenditure as well as exposure to work demands (e.g. fast-paced work) [22, 41] and psychosocial context at work (e.g. absence of management at night) [22, 57]. To counteract the effects of extended working hours or extended shifts, sufficiently long free time (e.g. breaks) for recovery activities (e.g. naps or exercise) during the work shift are needed, as well as positive psychosocial context at work (e.g. supportive colleagues) [22, 48, 49, 58–63].

Secondly, when the work schedule as a whole is considered, the durations of the working periods and free periods are important [22, 49, 64, 65]. Of additional importance is whether shifts are fixed or rotating [22, 32, 37]. When shifts rotate, the direction (forward or backward) and speed of rotation (number of identical consecutive shifts) are important [66, 67]. To counteract the effects of the work schedule, sufficiently long periods of non-work need to be scheduled between and after consecutive working days and shifts [22, 23, 25, 32, 65]. During non-work, activities can be pursued with potential for recovery and accommodation to a changed sleep/wake pattern [19, 22, 37, 40, 47, 68, 69]. Positive psychosocial factors related to the private life may also contribute to counteracting the negative effects of the work schedule [22, 40, 42, 49, 70].

#### Physiological processes

The aforementioned aspects of work (i.e. the time-of-day of work; the duration of a shift, working day, and schedule; fixed or rotating shifts; the direction and speed of rotation; work demands; and psychosocial context of work), may individually and combined lead to circadian disruption, sleep deprivation, and/or increased physiological activation. We propose that not all physiological processes are equally relevant for all types of nonstandard schedules: the presence of working time characteristics, work demands, and psychosocial context, determine which processes are activated. We further propose that these physiological processes are counteracted by recuperative processes during non-work: (re-)entrainment, restorative sleep, and complete recovery, respectively.

Firstly, unfavourable time-of-day, i.e. night work, early morning work, and late evening work, and disturbed daytime sleep are at odds with the body’s circadian rhythm and may cause circadian disruption [21, 31, 33, 42, 43, 45, 50, 51, 71, 72]. Circadian disruption has four operationalisations in the models in the literature review, which can be one or a combination of the following: a phase shift of a circadian rhythm relative to the day/night cycle, internal desynchronisation of different internal body rhythms, reduced rhythm amplitudes, and/or a desynchronisation between the central oscillators in the brain and those in peripheral tissues [19, 38, 42, 43, 45, 50]. For health maintenance, it is important to minimise circadian disruption [64, 66]. However, when circadian disruption does take place, the duration of disruption can be minimised by rapid re-entrainment of circadian rhythms to daytime wakefulness during the free period. Specifically for fixed schedules, rapid entrainment of the circadian rhythms to being awake during early morning, late evening, or night work could minimise the duration of circadian disruption. Activities that may function as ‘Zeitgebers’, such as timing of physical activity, as well as the presence of psychosocial private life factors, such as supportive and understanding family and friends, may also facilitate (re-)entrainment [20, 42, 73–75].

Secondly, working at an unfavourable time-of-day and changes in the sleep/wake pattern may lead to sleep deprivation [20, 33, 39, 40, 43]. Acute sleep loss that is associated with day-time sleep and disturbances in the distribution of sleep stages throughout sleep [20, 76, 77], may, with repeated work at unfavourable time-of-day, lead to an accumulation of sleep loss and chronic sleep deprivation [39, 40]. Restorative sleep during breaks or in between shifts seem to protect against such accumulation of sleep loss [39, 40, 58]. Restorative sleep may be facilitated by activities and psychosocial factors that influence physiological and cognitive de-arousal [40].

Thirdly, extended working hours, extended working weeks, high work demands with low control, and unfavourable psychosocial context at work, may lead to increased and/or sustained activation of the body’s physiological systems [23, 41, 53, 78]. This in turn may lead to sleep loss [12, 22, 79]. When increased activation is sustained until bedtime, this may delay and shorten sleep, thereby creating acute sleep loss, which over time may lead to sleep deprivation [39, 40]. To counteract the increased and/or sustained activation, complete recovery to a baseline level of activation is needed during non-work time [23, 25, 47]. Recovery can also be facilitated by activities and positive psychosocial factors that help to detach from work, e.g. physical exercise or positive experiences with family and friends [47, 69, 80].

Fourthly, in rotating shift schedules, a rapid forward rotation direction is more beneficial for health than a slow backward rotation direction [66, 67, 81, 82]. A forward rotation direction provides longer free periods between shifts, which gives more time to re-entrain circadian rhythms and to sleep. In the case of forward rotating shifts with extended working hours, unfavourable work demands, and/or psychosocial work context, the longer free periods also give more time to recover [66, 82]. A rapid speed of rotation entails that fewer consecutive shifts are worked compared to a slow rotation speed. A rapid speed may minimise circadian disruption and sleep deprivation by providing less time for disruption and sleep loss to take place [67].

To summarise, nonstandard schedules vary regarding working time characteristics and work characteristics, and it is the presence and the combination of these characteristics that determine which physiological processes are activated. For example, for shift schedules that include night work, early morning work, and/or late evening work, circadian disruption and sleep deprivation are relevant physiological processes. In day schedules with extended working hours and working weeks, physiological activation and sleep deprivation are relevant physiological processes. When these working time characteristics are combined, for example in shift schedules that include extended working hours (e.g. 12 h night shifts) or extended working weeks (e.g. 7 consecutive night shifts), all three physiological processes are relevant and may influence each other. All three physiological processes are also relevant for shift schedules that have unfavourable work demands and psychosocial context.

#### Health effects

When the physiological processes associated with nonstandard working schedules, i.e. circadian disruption, sleep deprivation, or activation, are not properly counterbalanced, they may lead to short-term health effects, such as sleepiness, fatigue, and shift work sleep disorder [5, 9, 13, 16]. These effects are often short lived and reversible with (re-)entrainment of the body’s circadian rhythm, restorative sleep, and complete recovery [21, 25, 39, 47]. The presence of these health effects may also negatively feed back onto the individual’s capacity to (re-)entrain, sleep well, and recover [22].

Long-term health effects are hypothesised to be a consequence of an inability to adapt to nonstandard working schedules, i.e. the inability to re-entrain the body’s circadian rhythms, sleep well, and recover. The continuous presence of desynchrony between work and biological rhythms, i.e. being active at a time when the body should be resting, could over time lead to increased vulnerability to disease [22, 32, 36, 43]. These diseases include obesity, metabolic syndrome, cardiovascular disease, diabetes mellitus type II, and cancer [14, 15, 18, 19, 21, 42, 43, 45, 83]. Some of these health effects may still be reversible, such as obesity and metabolic syndrome, if changes in lifestyle and coping behaviour are made. The presence of long-term health effects may negatively feed back onto the individual’s capacity to maintain a balance between the physiological processes [22].

#### Personal and situational factors

Personal and situational factors may influence the effects that work and non-work have on the physiological processes, as well as the effects that the physiological processes have on health. Personal factors are those related to the ability to adapt to working in nonstandard schedules, such as shift work tolerance, genetics, personality, coping strategies, behavioural and lifestyle changes, and shift work locus of control [19, 31, 42, 49, 50, 52–54, 84, 85]. Situational factors are those related to work and non-work that may fall outside of a worker’s direct control, such as working conditions, family composition, and housing conditions [37, 49].

## Discussion

A comprehensive model was developed on nonstandard working schedules and health that better reflects the diversity of schedules found in society and includes the health effects of rest periods. Central to the comprehensive model is the balance between physiological processes arising from working time characteristics and work characteristics that may lead to health complaints, and physiological processes related to non-work that protect from health complaints.

The fact that theoretical knowledge on the health effects of nonstandard working schedules has been deeply rooted in shift work literature was mirrored in the literature review: the far majority of models were on shift work, i.e. time-of-day (18 out of 22), while only one model was found on working time duration [41]. It is assumed that the theoretical background used by researchers studying working time duration has been rooted in theories on regular day work.

Three models were retrieved in the review that integrated the working time characteristics time-of-day and working time duration [22, 32]. The main weaknesses of the existing models on nonstandard working schedules and health are their shortcomings in describing the pathways to health. The two models described by Steinmetz and Schmidt [32] are oversimplified, and the model by Caruso et al. [22] is overly complex and fails to include the important pathway of circadian disruption for those working at night. In the new comprehensive model, three main recurrent physiological processes associating nonstandard working schedules with health are given (circadian disruption, sleep deprivation, activation) that were found throughout all models included in the review.

As only four models were found in the review that addressed working time duration, the theoretical perspective of recovery after work helped support the perspective of extended working hours in its integration with shift work theory. The comprehensive model developed in this paper drew from models on recovery after work by proposing a trade-off between work and non-work. This means that demands from the work arena, such as extended working hours, need to be counterbalanced by positive aspects from the non-work arena, such as sufficiently long breaks and time off. Attention for the trade-off between work and non-work is of specific importance in nonstandard working schedules, where the ratio between work and non-work is not fixed, and usually varies over time and between schedules. However, most likely a trade-off exists between negative and positive aspects of work as well; therefore, positive aspects of work can also be relevant for health maintenance. Similarly, most likely a trade-off exists between negative and positive aspects of non-work that also need to be balanced to maintain health.

### Strengths and limitations

This paper has several strengths. Firstly, where possible, we adhered to the PRISMA guidelines to systematically search, select, extract data, and report on existing frameworks, theories, and models from which the new comprehensive model was developed. Secondly, the model gives an overview of health-related risk factors of nonstandard working schedules and the complex pathways relating these schedules to health complaints. It thereby can function as a frame of reference and a starting point for creating a common language for researchers studying any form of nonstandard working schedules. Finally, this is the first model to specify the importance of both health damaging and health protective pathways related to nonstandard working schedules, illustrating that in order to prevent health problems not only damaging effects of work schedules should be considered but also the protective potential of breaks and leisure time.

This paper also has some limitations. Firstly, it cannot be ruled out that frameworks, theories, or models were missed by restricting the search to the databases Medline, PsycINFO, and EMBASE, and to the English language. However, these databases and the English language are expected to be the most important sources for relevant articles. Secondly, some of the assumed relationships in the new comprehensive model have not yet been verified by empirical studies. These pertain to the non-work side of the model and the protective physiological processes, e.g. the protective effect of (re-)entrainment on health, the optimal duration of free time, and the recovery effects of activities that shift workers pursue in their free time. Thirdly, like any model, the new comprehensive model is a simplification. It only includes overarching-terms for working time characteristics and not detailed descriptions of variables that fall under these overarching-terms, e.g. the proportion of night shifts (a time-of-day variable) or the proportion of extended night shifts (a working time duration variable) such as given by Härmä et al. [56]. The model also simplifies the health-related pathways so that they are relevant for general health outcomes; thereby the suggested pathway of vitamin D deficiency for cancer was excluded (see Table 2) [19]. Lastly, although working time control has been suggested as a means to improve recovery and restorative sleep and inclusion into the model could have advanced working time theory, it was excluded from the model to give alternative health maintenance recommendations for schedules that do not permit control over working time.

### Recommendations for future research

For future research, we recommend that the assumed relationships in the model will be empirically verified by summarizing available evidence in the scientific literature on nonstandard working schedules and health. Furthermore, newly conducted studies on the health effects of nonstandard working schedules should take a comprehensive approach and study both the individual as well as the combined effects of working time duration and time-of-day, work demands, and psychosocial work context in order to understand their relationship with health. In these studies, multiple outcome measures should be included on circadian adjustment, sleep, and on activation and recovery, corresponding to the assumed underlying mechanisms of health. In addition, attention should be paid to the contribution of non-work time and activities to understand how the duration of breaks, naps at work, or time off, as well as activities pursued in non-work time may contribute to (re-)entrainment, sleep, and recovery. In the end, this should lead to knowledge needed to define what ‘adequate rest’ is and to the optimal combination of these factors in (re-)designing working schedules. In this way, the model may serve as a tool for future research and practise in optimisation of nonstandard working schedules in order to maintain workers’ health.

## Conclusions

A comprehensive model on nonstandard working schedules and health was developed. The model adopts a comprehensive approach to working time characteristics and work characteristics as well as their related physiological pathways leading to health complaints (circadian disruption, sleep deprivation, and activation). The model further proposes that work needs to be counterbalanced by non-work factors during rest periods and their associated physiological pathways that are protective of health ((re-)entrainment, restorative sleep, and recovery). This model gives researchers a useful overview over the various risk factors and pathways associated with health that should be considered when studying any form of nonstandard working schedules.
